# Neutrophil-to-lymphocyte ratio as a potential biomarker in predicting in-stent restenosis: A systematic review and meta-analysis

**DOI:** 10.1371/journal.pone.0322461

**Published:** 2025-05-16

**Authors:** Paulus Parholong Siahaan, Wynne Widiarti, Pandit Bagus Tri Saputra, Rendra Mahardhika Putra, Mario D’Oria

**Affiliations:** 1 Faculty of Medicine, Universitas Airlangga, Surabaya, East Java, Indonesia; 2 Department of Cardiology and Vascular Medicine, Faculty of Medicine, Universitas Airlangga, Surabaya, East Java, Indonesia; 3 Department of Cardiology and Vascular Medicine, Dr Soetomo General Academic Hospital, Surabaya, East Java, Indonesia; 4 Division of Vascular and Endovascular Surgery, Department of Clinical Surgical and Health Sciences, University of Trieste, Trieste, Italy; Kasturba Medical College, Manipal Academy of Higher Education, INDIA

## Abstract

**Background:**

In-stent restenosis (ISR) remains a significant challenge despite advancements in percutaneous interventions, often leading to adverse clinical outcomes. The neutrophil-to-lymphocyte ratio (NLR) has emerged as a potential biomarker for predicting ISR, offering opportunities for improved risk stratification and treatment personalization. This systematic review and meta-analysis assess the predictive value of NLR in ISR, providing insights into its clinical applicability.

**Methods:**

Systematic literature search was conducted in scientific databases until 21st July 2024. Observational studies evaluating NLR in stented patients were included. Random effect meta-analysis and linear regression model were used to investigate odds ratios (OR) as predictor and linear dose-response relationship of ISR. Sensitivity and specificity of NLR to predict this outcome were pooled and a summary receiver operating characteristics (sROC) curve was generated. This study was already registered in the PROSPERO (ID: CRD42024555123).

**Results:**

15 studies with 3 889 patients were included. High NLR was associated with increased risk of ISR in coronary and non-coronary stenting [aOR = 1.61 (95%CI 1.14–2.25); aOR = 1.69 (95%CI 1.52–1.87)]. One unit increase of NLR is equal to 30% and 44% increased risk of ISR in coronary and non-coronary patients. Included studies showing NLR as a robust predictor of ISR with sensitivity and specificity of 70.5% (95%CI 60.1%-79.2%) and 74.1% (95%CI 56.7%-86.2%) for coronary stenting and 77.7% (95%CI 69.8%-84.0%) and 66.4% (95%CI 49.6%-79.8%) non-coronary stenting, with AUC of 0.77 (0.70–0.82) in the coronary and 0.79 (0.70–0.85) in the non-coronary sub-groups.

**Conclusion:**

In conclusion, NLR yields promising predictive and prognostic potentials in predicting ISR in coronary and non-coronary stents. Additionally, NLR appears to be more proficient in predicting early ISR compared to late ISR in both coronary and non-coronary stents.

## Introduction

Over the past decades, percutaneous vascular interventions have made remarkable progress. Despite these advancements, in-stent restenosis (ISR) remains a major clinical challenge, due to the proliferation of vascular smooth muscle cells and subsequent neointimal formation. These developments can impair stent effectiveness, leading to symptom recurrence and the need for further revascularization [1]. In non-coronary stenting, ISR has been reported in 18–40% of patients within the first year of treatment, especially in long and complex lesions [2]. In coronary stenting, the latest drug-eluting stents (DES) significantly reduce neointimal hyperplasia and the risk of ISR, improving the efficacy of percutaneous coronary intervention (PCI), with 50–70% of cases particularly occurring within the first month. This condition poses ongoing clinical challenges and increases the risk of major adverse cardiac events compared to PCI for new lesions [3,4].

Beyond the established cardiovascular risk factors, easily accessible laboratory parameters can enhance risk stratification for patients undergoing percutaneous vascular interventions. Inflammation plays a crucial role in ISR development and recent studies have highlighted the prognostic significance of inflammatory biomarkers, such as neutrophil-to-lymphocyte ratio (NLR) in predicting adverse cardiovascular events and procedural outcomes. NLR is cost-effective and widely available, making them particularly useful and applicable in both high-resource and low-resource healthcare settings. [5]. It is proven to be associated with adverse outcomes in various cardiovascular conditions. NLR shows promising potential as a predictor of ISR due to its correlation with the severity and activity of atherosclerotic and vascular disease as the fundamental causes of ISR. Although the link between various inflammatory markers, including NLR, has been explored, their predictive value in predicting ISR remains unclear [6]. This systematic review and meta-analysis aims to thoroughly evaluate the prognostic utility of NLR in predicting ISR in order to enhance risk stratification and contribute to a more personalized treatment strategies for patient management following both coronary and non-coronary stent implantation.

## Materials and methods

A protocol was registered in PROSPERO (CRD42024555123). This systematic review was conducted in accordance to the Cochrane Handbook for Systematic Reviews of Diagnostic Test Accuracy version 1.0.0 and followed the Preferred Reporting Items for a Systematic Review and Meta-Analysis (PRISMA) guideline, for reporting research merit and transparency.

### Search strategy

A systematic literature investigation was conducted on July 21^st^, 2024, across multiple scientific databases including PubMed, Scopus, Web of Science, ProQuest, and Cochrane, using a predefined set of keywords and synonyms based in MeSH terms and Boolean operators, listed in the Supplementary file. The process was done by two authors (PPS and WW) independently to screen for relevant articles according to the inclusion criteria and to assess the full texts subsequently. Manual searching was conducted to ensure comprehensive coverage. Any discrepancies were discussed with senior authors. Further details regarding the search strategy are summarized in S1 Table.

### Eligibility criteria

This study included cohort studies using predetermined inclusion criteria including: (1) patients with coronary or non-coronary stents, (2) reporting NLR values, (3) investigating the diagnostic accuracy of NLR in predicting ISR or assessed NLR as a prognostic factor, (4) written in English. The definition of ISR was more than 50% restenosis of the previously stented site [7]. Neutrophil lymphocyte ratio defined as the ratio of measured neutrophil and lymphocyte determined by each study. Records with inappropriate study design (case reports, case series, editorials, protocols, and conference abstracts), no available full-text articles, and literature not written in English. Further details regarding eligibility criteria are presented in S2 Table.

### Data extraction

From the included studies, we extracted the following data: first authors name and publication year, study characteristics such as study period, duration of follow-up, study design, country, and lab brand; patient characteristics such as population, sample size, age, sex, comorbidities, timing of blood workup (pre-procedural vs post-procedural), stent location (coronary vs non-coronary stent); and the investigated outcome, which were predictive test metric (true positive [TP], false negative [FN], true negative [TN], false positive [FP]). Whenever the included studies did not provide these metrics, we followed the University of Oxford Centre of Evidence-Based Medicine (CEBM) guideline [8], by transforming the available sensitivity, specificity, and the number of participants in the study to generate the sought variables. The duration of follow-up was further categorized into early ISR (≤1 year) and late ISR (>1 year) [6]. The coronary stent location refers to any stent implanted in the coronary arteries, while the non-coronary stent location refers to any stent implanted in the central and peripheral arteries. Two independent authors systematically extracted the relevant data using a pre-piloted form through Microsoft Excel® 2019 MSO. Any disagreements during the process were discussed through consensus with the senior author (S6 Table).

### Quality assessment

Two authors independently conducted the methodological quality assessment using the Quality in Prognosis Studies (QUIPS) tool [9] and Quality Assessment of Prognostic Accuracy Studies (QUAPAS) tool [10], which assessed six bias domains including study participation, study attrition, prognostic factor measurement, outcome measurement, study confounding, and statistical analysis and reporting. Details on each signaling question of the QUIPS tool are presented in S3 Table. Discrepancies between reviewers were resolved through discussion with senior reviewer. Studies were rated as having low, moderate, or high risk of bias in each domain, and an overall quality score was assigned. This comprehensive quality assessment ensured that only high-quality studies contributed to our final analysis, enhancing the reliability of our findings. The results of the quality assessment are detailed in S4 Table.

### Statistical analysis

All statistical analyses were performed using R software version 4.3.2 (Posit PBC, USA) with the “meta” and “mada” packages to estimate the pooled sensitivity and specificity using a bivariate meta-analysis. This analysis employed the Rutter and Gatsonis hierarchical summary receiver operating characteristic (HSROC) parameterization model [11]. Individual study estimates were visualized using Review Manager 5.4, which generated a summary receiver operating characteristics (sROC) curve visualizing the 95% confidence regions (CRs) and summary points while simultaneously obtaining the area under the curve (AUC). AUC values were interpreted as follows: 0.50–0.60 (failed), 0.60–0.70 (poor), 0.70–0.80 (fair), 0.80–0.90 (good), and ≥ 0.90 (excellent). To explore sources of heterogeneity, we performed subgroup analysis based on study region, sample size, study design, blood workup, follow-up period. For multivariate analysis, we summarized adjusted odds ratio to analyze the risk estimates and linear relationship between NLR and ISR using at random-effects meta-analysis mode. Heterogeneity was quantified using I^2^ and chi-square. To examine publication bias, funnel plots and Egger’s tests were conducted when the pooled analysis incorporated more than ten studies. The p-value < 0.05 was considered statistically significant in all analyses.

## Results

### Study selection

**Fig 1:**
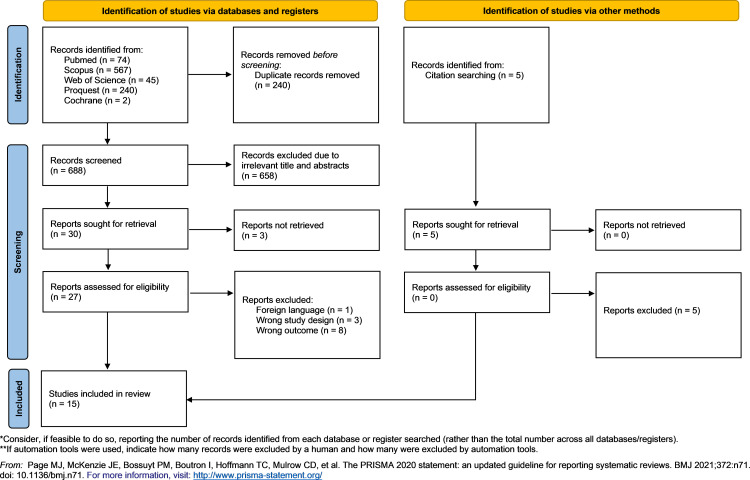
PRISMA flow diagram of the study selection process.

Initially, our study managed to collectively gather 950 studies upon electronic searching of the databases. Two hundred and forty duplicates were subsequently removed. In accordance with the predefined criteria, 680 articles were excluded during the title and abstract screening. Among the screened articles, three were excluded due to inappropriate study designs (review articles and conference abstracts) [12,13], written in non-English [14], and three due to irretrievable full text articles [15–17]. Further, eight articles were excluded due to reporting outcome of interest [18–25]. Finally, 15 studies were considered as eligible for further analysis for this study [6,26–39]. The PRISMA flow diagram recorded thoroughly the inclusion and exclusion of the articles (Fig 1 and S1 Fig).

### Study characteristics

**Fig 2:**
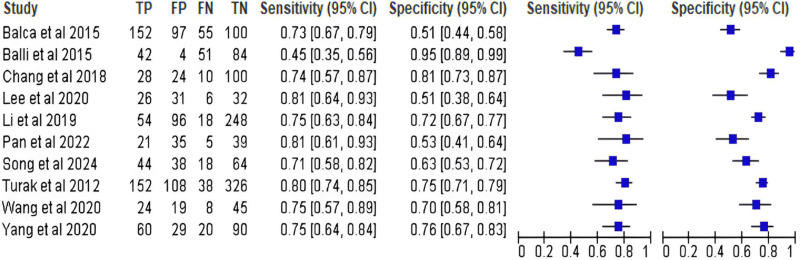
Forest Plot Illustrating the Study-specific Sensitivity and Specificity of the Included Studies in Predicting ISR.

A total of 3 889 patients from observational retrospective [26–28,30,34–36,39] and prospective [6,29,31–33] studies were included. These studies were conducted in Asia [6,26–28,31,34,35,37–39] and Europe [29,30,32,33,36]. Regarding stent location, eight of which are coronary stent procedures [28–30,32,34–36] and seven of which are non-coronary stent procedures [6,26,27,33,38–40], including stent in the femoropopliteal arteries, carotid arteries, superficial femoral artery, and intracranial arteries. Each individual study presented varying cut-off values to determine the optimal sensitivity and specificity. Details for studies characteristics were visualized in Fig 2 and S7 Table. The quality assessment for each individual study showed that all included articles had a low-to-moderate risk of bias (8 studies and 7 studies, respectively). Further details regarding baseline characteristics of included studies were summarized in S5 Table.

### Prognostic value and linear relationship of nlr in predicting coronary ISR

There were seven studies of 2 392 participants reporting data of risk for ISR. High value of NLR were linked to 60% increased odds of ISR (aOR = 1.60, 95%CI 1.14–2.25) (Fig 2), with a high level of heterogeneity (I^2^ = 88%) (S8b Table and S8 Fig). An aOR of 1.30 (95%CI 1.09–1.54) were found to be the value of NLR per unit in predicting ISR (S11 Table). The leave-one-out sensitivity analysis showed no significant reduction in heterogeneity and subgroup analysis failed to explore potential causes of heterogeneity. The summary and leave-one-out sensitivity analysis were visualized in S2 Fig. Publication bias analysis of Begg’s funnel plot and Egger’s regression test were not performed due to lack of required number of studies.

### Predictive value of nlr in predicting coronary ISR

To predict coronary ISR, six studies of 1 885 patients were considered as appropriate for analysis, and was found that NLR has a value of sensitivity and specificity of 70.5% (95%CI 60.1–79.2) and 74.1% (95%CI 56.7–86.2), respectively. The summary ROC curve (presented in Fig 4) showed pooled AUC of 0.77 (95%CI 0.70–0.82) and relatively narrow 95%CR. Other predictive measures analyzed were positive likelihood ratio (PLR) of 2.84 (1.75–4.56), negative likelihood ratio (NLR) of 0.41 (0.32–0.50), and diagnostic odds ratio (DOR) of 7.12 (4.02–11.50) (S8a Table and S10 Table).

**Fig 3:**
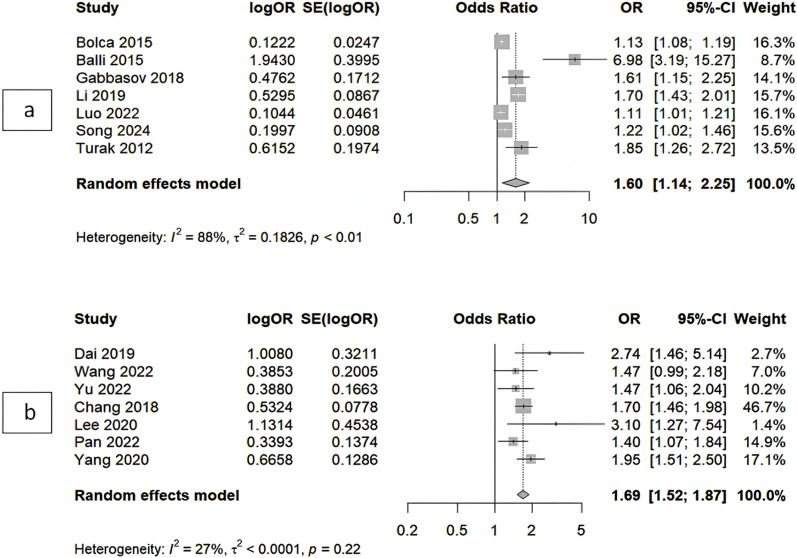
Forest Plot Illustrating Study-specific and Overall Adjusted Odds Ratio of NLR in Predicting Coronary (a) and Non Coronary (b) ISR.

**Fig 4:**
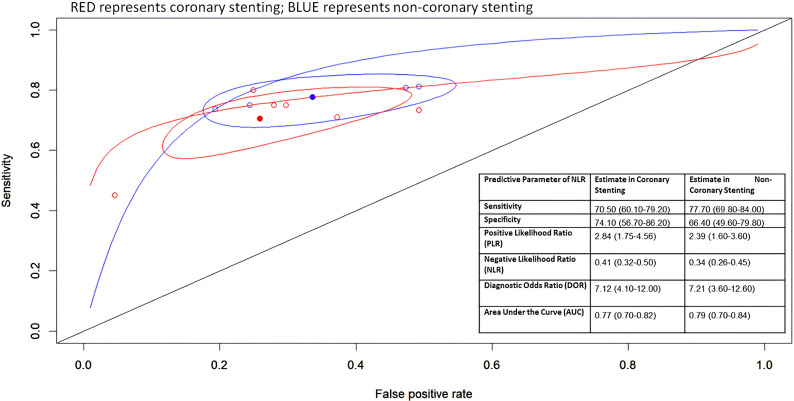
Summary receiver operator characteristic (sROC) curve of overall NLR for predicting ISR.

A sub-analysis was performed to evaluate how the parameter estimates of the variable influenced the predictor's performance, as shown in Table 1. NLR has a higher pooled sensitivity to predict late than early coronary ISR (early vs late 60.7% (95%CI 29.6–85.0) vs 73.4% (95%CI 68.5–77.9), P_sensitivity _= .156). Notably, NLR possesses higher specificity to predict early compared to late coronary ISR (early vs late 87.3% (95%CI 44.3–98.3) vs 62.3% (95%CI 49.1–73.9), P_specificity _= .118), although statistically insignificant. Additionally, NLR exhibits an AUC of 0.78 (95%CI 0.51–0.94) and 0.73 (95%CI 0.63–0.80), subsequently, to predict early and late coronary ISR. Study by Turak et al. (2012) [36], did not mention follow-up period specifically, therefore not included in the predictive follow-up period subanalysis. According to the stent type, NLR has a sensitivity of 67.6% (95%CI 45.2–84.1), specificity of 79.8% (95%CI 41.1–95.7), and AUC of 0.77 (95%CI 0.62–0.89) to predict ISR in DES sensitivity of 75.0% (95%CI 65.8–82.4), specificity of 71.8% (95%CI 67.2–76.0), and AUC of 0.78 (0.69–0.84).

**Table 1:** Summary of subgroup analyses of predictive tests.

Subgroup	Number of Studies	Sensitivity (95% CI)	Specificity (95% CI)	AUC (95%CI)	P_Sensitivity_	P_Specificity_
**CORONARY STENT**						
**Region (**S4 Fig)						
Asia [34,35,37]	3	73.5 (65.9-79.8)	69.0 (62.7-74.8)	0.77 (0.67-0.82)	0.579	0.519
Europe [29,30,36]	3	67.6 (45.2-84.1)	79.8 (41.1-95.7)	0.77 (0.62-0.89)		
**Sample Size (**S5 Fig)						
Small (n < 150) [37]	1	75.0 (57.0-89.0)	70.0 (58.0-81.0)	0.80 (0.71-0.89)	0.735	0.844
Large (n ≥ 150) [29,30,34–36]	5	69.9 (57.4-79.9)	75.1 (53.3-88.8)	0.78 (0.69-0.83)		
**Study Design (**S3 Fig)						
Cohort retrospective [30,34–37]	5	76.0 (71.7-79.9)	66.6 (57.3-74.7)	0.78 (0.71-0.82)	***0.000*** ^*^	***0.001*** ^*^
Cohort prospective [29]	1	45.5 (35.4-55.3	95.8 (88.5-98.3)	0.77 (0.70-0.83)		
**Follow Up Period (**S7 Fig)						
Early [29,37]	4	60.7 (29.6-85.0)	87.3 (44.3-98.3)	0.78 (0.53-0.94)	0.156	0.118
Late [30,34,35]	5	73.4 (68.5-77.9)	62.3 (49.1-73.9)	0.73 (0.64-0.80)		
**Stent Type**						
Bare Metal Stent [29,30,36]	3	67.6 (45.2-84.1)	79.8 (41.1-95.7)	0.77 (0.62-0.89)	0.574	0.689
Drug Eluting Stent [34,37]	2	75.0 (65.8-82.4)	71.8 (67.2-76.0)	0.78 (0.69-0.84)		
**NON-CORONARY STENT**						
**Region (**S4 Fig)						
Asia [6,27,38]	3	76.4 (67.6-83.5)	70.9 (52.8-84.2)	0.79 (0.69-0.86)	0.592	0.285
Europe [33]	1	81.3 (64.1-91.3)	50.8 (38.6-62.8)	0.63 (0.66-0.81)		
**Sample Size (**S6 Fig)						
Small (n < 150) [27,33]	2	81.0 (68.9-89.2)	51.8 (43.5-60.1)	0.68 (0.49-0.85)	0.342	***0.000*** ^*^
Large (n ≥ 150) [6,38]	2	74.6 (66.0-81.6)	78.1 (72.4-82.9)	0.83 (0.71-0.87)		
**Study Design (**S3 Fig)						
Cohort prospective [6,33,38]	3	76.6 (67.7-83.7)	70.5 (51.1-84.5)	0.79 (0.69-0.86)	0.675	0.366
Cohort retrospective [27]	1	80.1 (61.3-91.8)	52.7 (41.4-63.8)	0.67 (0.74-0.82)		
**Follow-Up Period (**S7 Fig)						
Early [6,38]	2	74.6 (66.0-81.6)	78.1 (72.4-82.9)	0.83 (0.72-0.87)	0.342	***0.000*** ^*^
Late [27,33]	2	81.0 (68.9-89.2)	51.8 (43.5-60.1)	0.68 (0.49-0.85)		

*p < 0.05 statistically significant

### Prognostic value and linear relationship of nlr in predicting non -coronary ISR

A total of 1 285 subjects from 7 studies were analyzed, showing in non-coronary stenting NLR has an aOR of 1.69 (95%CI 1.52–1.87), with a low heterogeneity between the studies (I^2^ = 27%) (Fig 3B). A linear relationship of per unit NLR with an aOR 1.44 (95%CI 1.20–1.73) for ISR (S9 Fig). Begg’s funnel plot and Egger’s regression test of publication bias were not performed due to a limited number of studies.

### Predictive value of nlr in predicting non-coronary ISR

Four studies were considered appropriate for analysis, resulting in 594 populations to be analyzed. The finding presented a pooled predictive estimate of: sensitivity, 77.7% (95%CI 69.8–84.0); specificity, 66.4% (95%CI 49.6–79.8); PLR: 2.39 (95%CI 1.60–3.60); NLR: 0.34 (95%CI 0.26–0.45); DOR: 7.21 (95%CI 3.60–12.60). The SROC curve visualized a relatively narrow 95%CR with an AUC value of 0.79 (95%CI 0.70–0.84).

Subgroup analysis indicated that in predicting early ISR in the non-coronary stenting, NLR possessed a sensitivity of 74.6% (95%CI 66.0–81.6), specificity of 78.1% (95%CI 72.4–82.9), and AUC of 0.83 (95%CI 0.71–0.87). For late ISR, the pooled measure was sensitivity of 81.0% (95%CI 68.9–89.2), specificity of 51.8% (95%CI 43.5–60.1), and AUC of 0.68 (95%CI 0.49–0.86). A statistically significant difference in predictive performance was observed from specificity (P_specificity_ < .001) rather than sensitivity (P_sensitivity_ = .342). Sub-analysis for stent type cannot be done due to inappropriate data provided by the studies.

## Discussion

In stent restenosis, defined as the re-narrowing of the more than 50% the previously stented vessel [41]. It remains as one of the most common and challenging problems for interventionists worldwide, despite advancements in stent technologies [42,43]. Predictive biomarkers are essential to facilitate risk stratification, enabling clinicians to implement timely and targeted interventions, in order to improve clinical outcomes [1,7].

### Coronary stenting

This study demonstrated that NLR provides a promising potential in predicting coronary ISR. In terms of coronary stenting, a high NLR has 1.6-fold of greater risk in having ISR, despite leave-one-out analysis and subgroup analysis cannot explained the potential cause of high heterogeneity, and an increase in per unit of NLR is equivalent of 30% increased odds of experiencing ISR. The result of this study aligns with previous studies, underscoring that ISR is a primarily atherosclerotic phenomenon characterized by neointimal hyperplasia within the stent [44–46]. Angkananard et al. (2018) reported similar results, showing that high NLR is associated with a 1.6-fold increased risk of acute coronary syndrome (ACS) [47].

Under physiological conditions, neutrophils exert pro-inflammatory effects by secreting cytokines, while lymphocytes display anti-inflammatory properties, highlighting the role of complementary immune pathways [48]. ISR is driven by endothelial injury from stent deployment, which triggers an inflammatory cascade that promotes vascular smooth muscle cell proliferation and extracellular matrix (ECM) deposition [49], leading to the development of neointimal hyperplasia [1,50]. The endothelial cells subsequently express adhesion molecules (e.g., ICAM-1, VCAM-1, and P-selectin) [51], resulting in the influx of the neutrophils and monocytes to the subendothelial space, thus accelerating neointimal growth [52]. Therefore, an increased neutrophil count can foresee the progression of neointimal development [38]. Lymphocytes, as immunomodulator, responding to physiological stress, including cortisol, indicating that NLR has a significant predictive value in this clinical context [13].

This theory is aligned with the result of this study, which indicates the promising performance of NLR in predicting ISR among stented patients. Numerous studies focusing on NLR also identify consistent results in predicting ISR, particularly in coronary stenting. The predictive performance of NLR for ISR is further supported by prior research, which has established NLR as a robust biomarker for coronary artery disease and adverse events following cardiovascular surgery [53,54]. Gordhanbhai et al., (2023) also demonstrated that NLR is beneficial in predicting severe stenosis of coronary arteries [55]. Köklü et al. (2016) also reported that NLR is a sufficient predictor of developing symptomatic plaque in patients with coronary artery disease [56]. Regardless of the type of stenting, this study demonstrate NLR has the same clinical benefit in both BMS and DES, although DES has shown to be more preferable in most clinical cases.

When considering the time interval to ISR since stent placement, the current finding suggested that NLR has better accuracy in predicting early compared to late coronary ISR, although statistically insignificant. This can be explained by the fact that during the acute phase of ISR, neutrophils and monocytes adhere to the site of the injured vascular tissue, accompanied by the deposition of fibrin and platelets. Macrophages and giant cells then gradually take over within weeks as the process progresses [57].

### Non-coronary stenting

On the other hand, NLR also showed potential in predicting ISR in patients undergoing non-coronary stenting. Higher NLR faced 1.7 times the fold of ISR occurrence. Linear dose response relationship demonstrated 40% chance of having ISR per increase in one-unit NLR. The present result showed NLR possesses favorable predictive value. Further analysis revealed the usage of NLR is superior and in predicting early non coronary ISR, reflected by the number of overall AUC, than late non coronary ISR.

A study by Russu et al. (2022) demonstrated that NLR to be valuable in predicting amputation rate and 12-month primary patency in PAD population [58]. In addition, Coelho et al. (2021) also demonstrated that higher NLR predicted a higher odd for 30-day mortality or amputation in patients with acute limb ischemia (ALI), as another form of atherosclerotic event [59]. These studies reinforce the relevance of NLR as a predictive marker for ISR across different vascular conditions, particularly in non-coronary stenting.

Similar to coronary stenting, inflammation is also a critical factor in the development of ISR in non-coronary stenting including peripheral and carotid arteries, involving early-stage neointimal hyperplasia following arterial injury, where neutrophils play a critical role through the release of myeloperoxidase, matrix metalloproteinase 9 (MMP-9), and free radicals, while certain lymphocyte subsets reduce inflammation. Following arterial stenting, platelet and fibrin accumulate around the stent, sparking acute inflammation initially, which transitions to a granulation tissue response involving neovascularization, smooth muscle cell migration, proliferation, and the gradual replacement of acute inflammatory cells with chronic ones [60]. Recent evidence suggests that the inflammatory response following vascular interventions differs by procedure type. Scalise et al. (2025) [61] found that carotid endarterectomy (CEA) elicits a stronger inflammatory reaction than carotid artery stenting (CAS), as evidenced by elevated levels of inflammatory biomarkers post-surgery. High levels of C-reactive protein (CRP) and fibrinogen were identified as predictors of early restenosis, emphasizing the critical role of inflammation in vascular healing and ISR.

Patients with in-stent restenosis (ISR) and a high neutrophil-to-lymphocyte ratio (NLR) are more likely to have soft plaque, which facilitates guide wire passage but is prone to rupture or dislodgement. Davies et al. (2010) found that soft plaque, combined with elevated high-sensitivity C-reactive protein (hsCRP), is a key predictor of ISR. Additionally, patients with coronary artery disease (CAD) and high NLR have increased inflammation and a higher risk of acute coronary events. During superficial femoral artery interventions, 3.8% of patients experienced distal embolization, likely due to soft plaque fragmentation from balloon angioplasty [62]. Therefore, the NLR could be an objective tool for predicting the complexity of ISR, assisting physicians in developing strategies to enhance operational efficiency and reduce distal embolization.

Notably, this predictive power of NLR persisted despite some patients receiving anti-inflammatory statin therapy [63]. It is speculated that NLR correlates with plaque stability, predicting risks like distal embolism during procedures. This theory suggests that patients with ISR and higher NLR may have softer plaques, which easier for guiding wires to navigate, but also prone to rupture or dislodge more easily [64]. Additionally, it is important to consider the role of omics science in the identification of biomarkers for ISR. Costa et al. (2024) [65] reported that advanced omics technologies have identified critical genetic and proteomic markers associated with restenosis, providing a precision medicine approach to vascular interventions in detecting inflammatory responses, plaque vulnerability, and restenosis risk [65].

This study has certain limitations. First, included studies were conducted in Asia and Europe, suggesting that our findings may not be broadly applicable to other populations and ethnicities. Second, there is a relative paucity of studies investigating the role of NLR among patients with non-coronary stents, warranting further research in this area. Third, a high heterogeneity was observed in this study, with only stent location and study region partially explaining the source of heterogeneity. However, to the best of our knowledge, this systematic review and meta-analysis is the first to evaluate the predictive value of NLR and ISR among both coronary and non-coronary stents. Additionally, our findings suggest that NLR is a promising biomarker that can be potentially utilized in the future, possessing a high predictive and prognostic value and supporting its potential integration into routine clinical practice for risk stratification and personalized management strategies.

## Conclusion

In conclusion, neutrophil-to-lymphocyte ratio (NLR) is a promising biomarker for predicting in-stent restenosis (ISR) in both coronary and non-coronary stent placements. Additionally, NLR appears to be more proficient in predicting early ISR compared to late ISR in both coronary and non-coronary stents. These findings highlight the clinical utility of NLR in guiding post-stent surveillance strategies, though further prospective studies are warranted to validate its prognostic accuracy and optimize its clinical application.

## Supporting information

S1 File Supplementary figures, tables, and other details supporting the systematic review and meta-analysis of the neutrophil-to-lymphocyte ratio (NLR) in predicting in-stent restenosis (ISR); including search strategy (S1 Table), eligibility criteria (S2 Table), QUIPS signalling questions (S3 Table), risk of bias assessments (S4a–S4b Tables), study inclusion & exclusion criteria (S5 Table), data sources (S6 Table), data extraction details (S7 Table), statistical results (S8a–S8b Tables), additional tables (S9–S11 Tables), and figures (S1–S9 Figures). (DOCX)
