# Supplementary material for: Neutrophil-to-lymphocyte ratio as a potential biomarker in predicting in-stent restenosis: A systematic review and meta-analysis
Source: PLoS One. 2025 May 16;20(5):e0322461. doi: 10.1371/journal.pone.0322461 (PMC12083799; doi:10.1371/journal.pone.0322461)

**SUPPLEMENTARY MATERIALS**

**Neutrophil-to-lymphocyte Ratio as a Potential Biomarker in Predicting In-stent Restenosis: A Systematic Review and Meta-Analysis**

**S1 Table. Search Strategy.** Keywords and search terms used for database searches.

**S2 Table. Study Eligibility Criteria.** Inclusion and exclusion criteria based on PICOTS framework.

**S3 Table. QUIPS Tool Signalling Questions.** Signalling questions used to assess risk of bias across six domains.

**S4a Table. Risk of Bias Assessment of Included Studies.** Result of Domain-based QUIPS risk of bias ratings.

**S4b Table. Risk of Bias Assessment of Included Studies (Cont’d).** Additional detailed ratings per study.

**S5 Table. Identified Studies from the Literature Search.** Summary of included and excluded studies with rationale.

**S6 Table. Primary Research Sources.** Study identifiers, extractors, and eligibility details.

**S7 Table. Data Extraction from Primary Studies.** Study characteristics, stent location, and follow-up data.

**S8a Table. Predictive Accuracy Data.** Sensitivity, specificity, AUC, and blood work-up timing.

**S8b Table. Prognostic Test Data.** Adjusted odds ratios and covariates from multivariate analyses.

**S9 Table. Baseline Characteristics of Included Studies.** Patient demographics and comorbidity profiles.

**S10 Table. Outcomes of NLR Predictive Value for ISR.** Predictive performance of NLR after stent implantation.

**S11 Table. NLR and Prognosis of ISR.** Association between NLR and ISR prognosis.

**S1 Figure. PRISMA Checklist.** Flowchart showing article selection process.

**S2 Figure. Leave-One-Out Sensitivity Analysis.** Forest plot for coronary ISR prediction robustness.

**S3 Figures. Subgroup Analysis Forest Plots According to Study Design.** Stratified plots according to study design.

**S4 Figures. Subgroup Analysis Forest Plots According to Region.** Stratified plots according to region or location of study.

**S5 Figures. Subgroup Analysis Forest Plots According to Dose Category.** Stratified plots according to dose category.

**S6 Figures. Subgroup Analysis Forest Plots According to Sample Size.** Stratified plots according to sample size.

**S7 Figures. Subgroup Analysis Forest Plots According to Follow-up Period.** Stratified plots according to follow-up period.

**S8 Figures. Linear Dose-Response Relationships between NLR and Coronary ISR.** Graphical trends of NLR and ISR risk in coronary stents.

**S9 Figures. Linear Dose-Response Relationships between NLR and Non-coronary ISR.** Graphical trends of NLR and ISR risk in non-coronary stents.

**S1 Table. Search Strategy**

| **Database** | **Keywords** | **First Hit** | **Search Time** |
| --- | --- | --- | --- |
| *Pubmed* | (NLR OR "neutrophil-to-lymphocyte" OR (neutrophil* AND lymphocyt* AND ratio*)) AND ("Coronary restenosis"[Mesh] OR "Graft Occlusion, Vascular"[Mesh] OR "in-stent restenosis" OR "in-stent re-stenosis" OR "intra-stent restenosis" OR "intra-stent re-stenosis" OR "in-segment restenosis" OR "in-segment re-stenosis" OR ISR OR restenos* OR re-stenos* OR reocclus* OR re-occlus*) | 74 | June 10, 2023  (09:02, GMT+7) |
| *Scopus* | (NLR OR "neutrophil-to-lymphocyte") AND (restenosis OR re-stenosis OR reocclusion OR re-occlusion) | 567 | June 10, 2023  (09:04, GMT+7) |
| *Web of Science* | (NLR OR "neutrophil-to-lymphocyte") AND (restenosis OR re-stenosis OR reocclusion OR re-occlusion) | 45 | June 10, 2023  (09:05, GMT+7) |
| *Proquest* | (NLR OR "neutrophil-to-lymphocyte") AND (restenosis OR re-stenosis OR reocclusion OR re-occlusion) | 240 | June 10, 2023  (09:07, GMT+7) |
| *Cochrane* | (NLR OR "neutrophil-to-lymphocyte" OR (neutrophil* AND lymphocyt* AND ratio*)) AND ("Coronary restenosis"[Mesh] OR "Graft Occlusion, Vascular"[Mesh] OR "in-stent restenosis" OR "in-stent re-stenosis" OR "intra-stent restenosis" OR "intra-stent re-stenosis" OR "in-segment restenosis" OR "in-segment re-stenosis" OR ISR OR restenos* OR re-stenos* OR reocclus* OR re-occlus*) | 2 | June 10, 2023  (09:09, GMT+7) |

**S2 Table. Study Eligibility Criteria.**

The authors developed a framework of ‘Population, Index prognostic factor, Comparator prognostic factor, Outcome, Timing, Settings’ (PICOTS), adapted from guideline by Riley et al, to thoroughly define the objectives of our meta-analysis.

| **Item** | **Definition** |
| --- | --- |
| **P**opulation | Patients with implanted stents |
| **I**ndex prognostic factor | Neutrophil-Lymphocyte Ratio |
| **C**omparator prognostic factor | None |
| **O**utcome | In-Stent Restenosis (Predictive value, linear relationship, odds ratio) |
| **T**iming | Prognostic factor: at any point  Outcome: at any point |
| **S**etting | To obtain prognostic information about patients; this information guide clinicians for risk stratification in patients with implanted stents. |

**S3 Table. QUIPS Tool Signalling Questions**

The included studies were investigated for risk of bias using a tailored version of the Quality in

Prognosis Studies (QUIPS) tool, that assessed across six bias domains including study participation, study attrition, prognostic factor measurement, outcome measurement, and study confounding, and statistical analysis and reporting. Details on each signaling question of the QUIPS tool are presented in Table S1.

| **Signaling question** | **Issues to consider for judgment** |
| --- | --- |
| **High:** Most items are answered with ‘no’**; Low:** Most items are answered with ‘yes’**; Unclear:** Most items are answered with ‘unclear’  **Note:** A single ‘no’ answer may potentially result in high risk of bias, depending on study specifics | |
| **Study Participation** | |
| a. Description of source of population or population of interest | Is the source population for patients with coronary artery disease clearly described? |
| b. Adequate description of sampling frame and recruitment | Are the methods of sampling and potential biases in participant selection described (i.e., no selective sampling)? |
| c. Adequate description of recruitment period | Is the period of recruitment clearly defined? |
| d. Adequate description of place of recruitment | Is the place of recruitment clearly defined? |
| e. Adequate description of inclusion and exclusion criteria | Are the definitions of patients with coronary artery disease and other inclusion and exclusion criteria clearly described? |
| f. Adequate participation in the study by eligible individuals | Is the proportion of patients participating in the study adequate? |
| g. Description of baseline study sample | Are the baseline characteristics of the patients clearly described? |
| **Study Attrition** | |
| a. Adequate response rate for study participants | Was the proportion of participants completing the study with outcome data satisfactory (less than 10% missing)? |
| b. Description of attempts to collect information on participants who dropped out | Are the efforts to collect information from participants who dropped out of the study clearly documented? |
| c. Reasons for loss to follow-up provided | Are the reasons for participants dropping out of the study provided (e.g., transferred to another hospital, withdrew consent)? |
| d. Adequate description of participants lost to follow up | Are the key characteristics (e.g., age, sex, initial NLR values) of participants who dropped out described in detail? |
| e. No important differences between participants who completed the study and those who did not | Are any differences between participants who completed the study and those who did not documented as not significant, or is there sufficient information provided to assess whether the differences were significant? |
| **Prognostic Factor Measurement** | |
| a. A clear definition or description of prognostic factor measurement is provided | Is the method for obtaining the neutrophil-to-lymphocyte ratio clearly defined (e.g., automated blood count)? |
| b. Method of prognostic factor measurement is adequately valid and reliable | Is the method of measuring NLR adequately valid and reliable, such as standardized laboratory procedures? |
| c. Continuous variables reported or appropriate cut points used | Are continuous variables reported directly, or are standard categories or cut-off points used to classify NLR values? |
| d. Method and setting of prognostic factor measurement is the same for all study participants | Is the method and setting of measuring NLR the same for all study participants, ensuring uniformity across the sample? |
| e. Adequate proportion of the study sample has complete data prognostic factor measurement | Is the proportion of the study sample with complete NLR data adequate for analysis? |
| f. Appropriate methods of imputation was used for missing data | Are appropriate methods used for imputing missing NLR data, ensuring that analysis remains robust despite gaps in data? |
| **Outcome Measurement** | |
| a. Clear definition of the outcome provided | Measurement of poor composite outcome, mortality, or severity criteria should be clearly defined |
| b. Method of outcome measurement used is adequately valid and reliable | Method of outcome ascertainment is valid and reliable: based on events reported on medical records or valid severity criteria |
| c. Method and setting of outcome measurement is the same for all study participants | Measurement of poor composite outcome, severity, or mortality should be the same for all study participants |
| **Study Confounding** | |
| a. Important confounders are measured | Important confounders are age, sex, and comorbidities |
| b. Clear definitions of the important confounders measured are provided | Definition and measurement of confounders are clearly described |
| c. Measurement of confounders are adequately valid and reliable | Measurement of confounders are valid and reliable |
| d. Same method and setting of confounding measurement in all study participants | Measurements of confounders are the same for all study participants |
| e. Appropriate methods are used if imputation is | Methods to impute missing confounder data, if any, are appropriate and clearly described |
| f. Important potential confounders are accounted for in the study design | Strategies to account for potential confounders are described in the methods section of the study |
| g. Important potential confounders are  accounted for in the  analysis | Important confounders are accounted for in multivariable logistic regression and Cox proportional hazards models |
| **Statistical Analysis and Reporting** | |
| a. Sufficient presentation of data to assess the adequacy of the analysis | Mean or median values of variables are appropriately reported with standard deviations or interquartile range, results are properly reported with confidence intervals or standard errors |
| b. Strategy for model building is appropriate and based on a conceptual framework or model | N/A: Since the research question focuses on only one biomarker, model building strategies or conceptual framework are not expected |
| c. The selected statistical model is adequate for the design of the study | Univariate and multivariate logistic regression or Cox proportional hazard model are used as appropriate |
| d. No selective reporting of results | N/A: NLR as the prognostic factor of interest and its relation to severity or mortality should be reported, or else study would be excluded |
| **Overall Risk of Bias** | **Low**: Low risk of bias in all domains or moderate risk of bias in up to one domain;  **High**: High risk of bias in one or more domains or moderate risk of bias in most domains;  **Moderate**: All cases not fulfilling criteria for either low or high risk of bias |

**S4a Table. Risk of Bias Assessment of Included Studies**


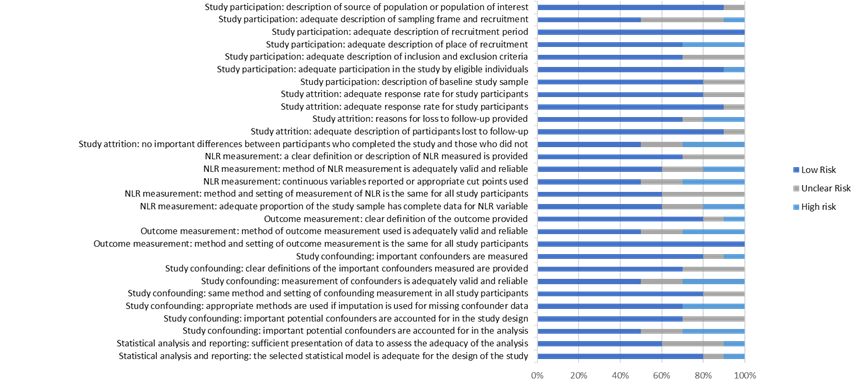


**S4b Table. Risk of Bias Assessment of Included Studies (Cont’d).**

|  | Statistical analysis and reporting: the selected statistical model is adequate for the design of the study | Statistical analysis and reporting: sufficient presentation of data to assess the adequacy of the analysis | Study confounding: important potential confounders are accounted for in the analyses | Study confounding: important potential confounders are accounted for in the study design | Study confounding: appropriate methods are used if imputation issued for missing confounder data | Study confounding: same method and setting of confounding measurement in all study participants | Study confounding: measurement of confounder is adequately valid and reliable | Study confounding: clear definitions of the important confounders measured are provided | Study confounding: important confounders are measured | Outcome measurement: method and setting of outcome measurement is the same for all study participants | Outcome measurement: method of outcome measurement used is adequately valid and reliable | Outcome measurement: clear definition of the outcome provided | NLR measurement: adequate proportion of the study sample has complete data for NLR variable | NLR measurement: method and setting of measurement of NLR is the same for all study participants | NLR measurement: continous variable reported or appropriate cut points used | NLR measurement: methods of NLR measurement is adequately valid and reliable | NLR measurement: a clear definition or description of NLR measured is provided | Study attrition: no important differences between participants who completed the study and those who did not | Study attrition: adequate description of participants lost to follow-up | Study attrition: reasons for loss to follow-up provided | Study attrition: adequate response rate for study participants | Study attrition: adequate response rate for study participants | Study participation: description of baseline study sample | Study participation: adequate participation in the study by eligible individuals | Study participation: adequate description of inclusion and exclusion criteria | Study participation: adequate description of place of recruitment | Study participation: adequate description of recruitment period | Study participation: adequate description of sampling frame and recruitment | Study participation: description of source of population or population of interest |
| --- | --- | --- | --- | --- | --- | --- | --- | --- | --- | --- | --- | --- | --- | --- | --- | --- | --- | --- | --- | --- | --- | --- | --- | --- | --- | --- | --- | --- | --- |
| Yang et al., 2020 |  |  |  |  |  |  |  |  |  |  |  |  |  |  |  |  |  |  |  |  |  |  |  |  |  |  |  |  |  |
| Chang et al., 2018 |  |  |  |  |  |  |  |  |  |  |  |  |  |  |  |  |  |  |  |  |  |  |  |  |  |  |  |  |  |
| Lee et al.,  2020 |  |  |  |  |  |  |  |  |  |  |  |  |  |  |  |  |  |  |  |  |  |  |  |  |  |  |  |  |  |
| Turak et al., 2012 |  |  |  |  |  |  |  |  |  |  |  |  |  |  |  |  |  |  |  |  |  |  |  |  |  |  |  |  |  |
| Balli et al., 2015 |  |  |  |  |  |  |  |  |  |  |  |  |  |  |  |  |  |  |  |  |  |  |  |  |  |  |  |  |  |
| Bolca et al., 2015 |  |  |  |  |  |  |  |  |  |  |  |  |  |  |  |  |  |  |  |  |  |  |  |  |  |  |  |  |  |
| Dai et al.,  2019 |  |  |  |  |  |  |  |  |  |  |  |  |  |  |  |  |  |  |  |  |  |  |  |  |  |  |  |  |  |
| Li et al.,  2019 |  |  |  |  |  |  |  |  |  |  |  |  |  |  |  |  |  |  |  |  |  |  |  |  |  |  |  |  |  |
| Gabbasov et al., 2018 |  |  |  |  |  |  |  |  |  |  |  |  |  |  |  |  |  |  |  |  |  |  |  |  |  |  |  |  |  |
| Wang et al., 2022 |  |  |  |  |  |  |  |  |  |  |  |  |  |  |  |  |  |  |  |  |  |  |  |  |  |  |  |  |  |
| Luo et al., 2022 |  |  |  |  |  |  |  |  |  |  |  |  |  |  |  |  |  |  |  |  |  |  |  |  |  |  |  |  |  |
| Yu et al., 2022 |  |  |  |  |  |  |  |  |  |  |  |  |  |  |  |  |  |  |  |  |  |  |  |  |  |  |  |  |  |
| Pan et al., 2022 |  |  |  |  |  |  |  |  |  |  |  |  |  |  |  |  |  |  |  |  |  |  |  |  |  |  |  |  |  |
| Wang et al., 2020 |  |  |  |  |  |  |  |  |  |  |  |  |  |  |  |  |  |  |  |  |  |  |  |  |  |  |  |  |  |
| Song et al., 2024 |  |  |  |  |  |  |  |  |  |  |  |  |  |  |  |  |  |  |  |  |  |  |  |  |  |  |  |  |  |

**S5 Table. Identified Studies from the Literature Search.**

| **No.** | **Authors’ ID** | **Status** | **Rationale for Exclusion** |
| --- | --- | --- | --- |
| 1 | Bao et al., 2020 | Excluded | No outcome available for NLR |
| 2 | Balli et al., 2015 | Included | N/A |
| 3 | Bolca et al., 2015 | Included | N/A |
| 4 | Chan et al., 2014 | Excluded | Not reporting ISR as the outcome specifically (Outcome of interest: Primary patency) |
| 5 | Chang et al., 2018 | Included | N/A |
| 6 | Chernyak et al., 2018 | Excluded | Irretrievable Full Text |
| 7 | Dai et al., 2019 | Included | N/A |
| 8 | Gabbasov et al., 2018 | Included | N/A |
| 9 | Jiang et al., 2021 | Excluded | Irretrievable Full Text |
| 10 | Lee et al., 2020 | Included | N/A |
| 11 | Li et al., 2017 | Excluded | No outcome available for NLR (Reporting NLR changes only) |
| 12 | Li et al., 2019 | Included | N/A |
| 13 | Luo et al., 2022 | Included | N/A |
| 14 | Nakazawa et al., 2017 | Excluded | No outcome available for NLR (Reporting Neutrophil and Lymphocyte separately) |
| 15 | Pan et al., 2023 | Included | N/A |
| 16 | Shames et al., 2019 | Excluded | Published not in English |
| 17 | Song et al., 2024 | Included | N/A |
| 18 | Tanidi et al., 2016 | Excluded | Not reporting ISR as outcome |
| 19 | Turak et al., 2012 | Included | N/A |
| 20 | Wang et al., 2022 | Included | N/A |
| 21 | Wang et al | Included | N/A |
| 22 | Yang et al | Included | N/A |
| 23 | Yu et al | Included | N/A |
| 24 | Zheng et al., 2018 | Excluded | Irretrievable Full Text |
| 25 | Zeng et al., 2021 | Excluded | No outcome available for NLR |
| 26 | Zhen et al., 2019 | Excluded | Not reporting ISR as outcome |

**S6 Table. Primary Research Sources**

| **No** | **Authors’ ID** | **Extractors (Date)** | **Status** |
| --- | --- | --- | --- |
| 1 | Balli et al., 2015 | Paulus (August 1^st^ 2024) | Eligible |
| 2 | Bolca et al., 2015 | Paulus (August 1^st^ 2024) | Eligible |
| 3 | Chang et al., 2018 | Paulus (August 1^st^ 2024) | Eligible |
| 4 | Dai et al., 2019 | Paulus (August 1^st^ 2024) | Eligible |
| 5 | Gabbasov et al., 2018 | Paulus (August 1^st^ 2024) | Eligible |
| 6 | Lee et al., 2020 | Paulus (August 1^st^ 2024) | Eligible |
| 7 | Li et al., 2019 | Wynne (August 1^st^ 2024) | Eligible |
| 8 | Luo et al., 2022 | Wynne (August 1^st^ 2024) | Eligible |
| 9 | Pan et al., 2023 | Wynne (August 1^st^ 2024) | Eligible |
| 10 | Song et al., 2024 | Wynne (August 1^st^ 2024) | Eligible |
| 11 | Turak et al., 2012 | Paulus (August 2^nd^ 2024) | Eligible |
| 12 | Wang et al., 2022 | Paulus (August 2^nd^ 2024) | Eligible |
| 13 | Wang et al | Paulus (August 2^nd^ 2024) | Eligible |
| 14 | Yang et al | Wynne (August 2^nd^ 2024) | Eligible |
| 15 | Yu et al | Wynne (August 2^nd^ 2024) | Eligible |

**S7 Table. Data Extraction from Primary Studies**

| **Author** | **Study Period** | **Follow-up Period** | **Study Design** | **Study Location** | **Laboratorium Brand** | **Population** | **Sample Size (Man %)** | **Age** | **Comorbidities** | | | | | **Procedure**  **(Type of Stent)** | **Stent Location** |
| --- | --- | --- | --- | --- | --- | --- | --- | --- | --- | --- | --- | --- | --- | --- | --- |
|  |  |  |  |  |  |  |  |  | **HT (%)** | **DM (%)** | **Dyslipidemia (%)** | **AF (%)** | **Smoking (%)** |  |  |
| Balli et al et al., 2015 | January 2010 - December 2012 | ISR (+): 9.6 ± 3.4  ISR (-): 8.9 ± 3.9 | Cohort Prospective* | Turkey | NA | Stable CAD patients with true bifurcation lesion | 181 (61.6) | ISR: 55.3 ± 10.0, non ISR: 56.4 ± 12.4 | ISR: 52.3  No ISR: 61.3 | ISR: 38.6  No ISR: 22.6 | ISR: 70.5  No ISR: 73.1 | NA | NA | Percutaneous Coronary Intervention  (Bare-metal stent) | Bifurcation area of the coronary arteries |
| Bolca et al., 2015 | January 2008 - December 2012 | 14 (6-60) | Cohort Retrospective | Turkey | Coulter LH 780 Hematology Analyzer; Beckman Coulter Ireland Inc., Mervue, Galway, Ireland | ST-segment elevation myocardial infarction (STEMI) patients and had undergone primary PCI | 404 (66.34) | Tertile 1: 14.5 ± 1.2  Tertile 2: 14.6 ± 1.2  Tertile 3: 14.4 ± 1.1 | Tertile 1: 45.0  Tertile 2: 39.0  Tertile 3: 44.0 | Tertile 1: 22.0  Tertile 2: 25.0  Tertile 3: 22.0 | Tertile 1: 36.0  Tertile 2: 30.0  Tertile 3: 30.0 | NA | Tertile 1: 35.0  Tertile 2: 50.0  Tertile 3: 61.0 | Percutaneous Coronary Intervention  (Bare-metal stent) | Coronary arteries (Left circumflex artery, left anterior descending artery, right coronary artery) |
| Chang et al., 2018 | January 2012 - December 2014 | 12 months | Cohort Prospective | China | Automated Blood Cell Counter (Model XE2100; Sysmex Co, Kobe, Japan) | Femoropopliteal chronic total occlusions patients | 180 (78) | ISR: 64.1 ± 11.0, Non-ISR: 68.0 ± 11.8 | ISR: 60.5  No ISR: 60.5 | ISR: 78.9  No ISR: 41.1 | ISR: 84.2  No ISR: 54.0 | NA | ISR: 44.7  No ISR: 47.6 | Percutaneous Coronary Intervention  (Bare-metal stent) | Femoral and popliteal artery |
| Dai et al., 2019 | March 2004 - December 2016 | 14.6 ± 19.1 (range:  0.7-120.7) months | Cohort Prospective | China | Automated Blood Analyzer (Sysmex XE-2100, Kobe, Japan) | Carotid artery stenosis | 295 (85.8) | ISR: 66.6 ± 7.1, non ISR: 66.9 ± 7.6 | 82.8 | 37.0 | 44.0 | NA | 39.2 | Carotid angioplasty and stenting  (Self-expandable stent) | Carotid artery |
| Gabbasov et al., 2018 | January 2012 - December 2015 | 6-12 months | Cohort Prospective | Russia | NA | Patients With Diabetes  Having Stable Coronary Artery Disease | 126 | DM: 62.4 (9.5)  Non-DM: 62.2 (11.3) | DM: 98.2  Non-DM: 91.2 | NA | DM: 100.0  Non-DM: 98.6 | NA | DM: 50.9  Non-DM: 47.9 | Percutaneous Coronary Intervention  (Drug-eluting stent) | Coronary arteries (Left circumflex artery, left anterior descending artery, right coronary artery) |
| Lee et al., 2020 | NA | 24 months | Cohort Prospective | Austria | NA | Infrainguinal artery stenosis of PAD with Rutherford stage 2-3 patients | 95 (60) | 65 (58-74) | 92.6 | 35.8 | 93.7 | NA | 44.2 | Infrainguinal angioplasty with stent  Implantation  (N/A) | Superficial femoral artery |
| Li et al., 2019 | January 2010 - April 2013 | 14.4 ± 3.3 months | Cohort Retrospective | China | Sysmex XS 500i auto analyzer (Sysmex,  Kobe, Japan) | Coronary chronic total occlusion (CTO) | 416 | ISR: 57.8 ± 10.5, non ISR: 60.2 ± 11.7 | CTO: 58.8  Non-CTO: 45.0 | CTO: 37.5  Non-CTO: 34.4 | CTO: 60.0  Non-CTO: 50.6 | NA | CTO: 58.1  Non-CTO: 34.4 | Percutaneous coronary intervention  (Drug-eluting stent) | Coronary arteries (Left circumflex artery, left anterior descending artery, right coronary artery) |
| Luo et al., 2022 | January 2017 and December 2020 | 6-24 months | Cohort Retrospective | China | NA | Patients with coronary artery disease | 477 (79) | ISR: 63(55.7)  non-ISR: 61(54.7) | ISR: 48.9  No ISR: 49.2 | ISR: 19.1  No ISR: 15.1 | NA | NA | ISR: 62.8  No ISR: 55.9 | Percutaneous coronary intervention  (Drug-eluting stent) | Coronary arteries (Left circumflex artery, left anterior descending artery, right coronary artery) |
| Pan et al., 2023 | January 2017 and December 2019 | Every 3-6 months (1, 3, 6 months, and so on) | Cohort Retrospective | China | Model XE2100; Sysmex Co, Kobe, Japan | Femoropopliteal arterial disease | 120 (78) | 70.0 ± 8.3 | Non-restenosis: 70.3  Early restensosis: 55.0  Late restenosis: 65.4 | Non-restenosis: 59.5  Early restensosis: 80.0  Late restenosis: 57.6 | NA | NA | Non-restenosis: 45.9  Early restensosis: 20.0  Late restenosis: 50.0 | Drug-coated balloon (DCB) angioplasty | Femoral and popliteal artery |
| Song et al., 2024 | January 2017 and January 2022 | 24 months | Cohort Retrospective | China | NA | Severe kidney disease (eGFR < 30 ml/min 1.73m) receiving coronary stent placement | 164 | ISR: 72 (70.6)  Non-ISR: 35 (56.5) | ISR: 95.2  Non-ISR: 85.3 | ISR: 67.4  Non-ISR: 54.9 | ISR: 87.1  Non-ISR: 87.3 | ISR: 6.5  Non-ISR: 6.9 | ISR: 22.6  Non-ISR: 33.3 | Percutaneous coronary intervention  (N/A) | Coronary arteries (Left circumflex artery, left anterior descending artery, right coronary artery) |
| Turak et al., 2012 | February 2008 - June 2010 | N/A | Cohort Retrospective | Turkey | Automated blood cell counter (ADVIA 2120i Hematology System, Siemens Healthcare Diagnostics, Deerfield, Illinois) | Stable or Unstable Angina Pectoris Patients | 624 (72) | 60.5 ± 10.2 | Tertile 1: 53.0  Tertile 2: 59.0  Tertile 3: 62.0 | Tertile 1: 28.0  Tertile 2: 30.0  Tertile 3: 35.0 | NA | NA | Tertile 1: 37.0  Tertile 2: 43.0  Tertile 3: 55.0 | Percutaneous Coronary Intervention  (Bare-metal stent) | Coronary arteries (Left circumflex artery, left anterior descending artery, right coronary artery) |
| Wang et al., 2022 | May 2016 to December 2020 | 12 months | Cohort Retrospective | China | NA | Lower extremity arterial ischemia symptoms and Rutherford grade 3-5 | 117 (66) | 67.0 (59.0, 71.0) | 41.0 | 65.0 | NA | NA | 10.3 | Drug-coated balloon angioplasty | Femoral and popliteal artery |
| Wang et al | January 2016 and January 2019 | 6-15 months | Cohort Retrospective | China | NA | Coronary artery disease | 96 (71) | 65.3 ± 8.4 | NA | NA | NA | NA | NA | Percutaneous coronary intervention  (Drug-eluting stent) | Coronary arteries (Left circumflex artery, left anterior descending artery, right coronary artery) |
| Yang et al | March 2015 - July 2018 | 12 months | Cohort Prospective | China | Auto-Analyzer (Model XE2100; Sysmex Co, Kobe, Japan) | Lower extremity arteriosclerosis obliterans patients | 199 (77.9) | ISR: 68.72 ± 11.37, Non-ISR: 70.59 ± 9.52 | ISR: 77.5  No ISR: 82.4 | ISR: 42.5  No ISR: 32.6 | ISR: 80.0  No ISR: 67.8 | NA | ISR: 35.0  No ISR: 30.7 | Percutaneous transluminal stent implantation  (Self-expanding balloon) | Superficial femoral artery |
| Yu et al | June 2012 to September 2019 | 48 months | Cohort Retrospective | China | Automatic particle counters at hematology laboratories in Tiantan Hospital | Symptomatic intracranial atherosclerotic stenosis | 279 | 57.73 ± 8.73 | ISR: 77.5  Intermediate ISR: 76.7  No ISR: 82.4 | ISR: 42.5  Intermediate ISR: 37.1  No ISR: 32.7 | ISR: 80.0  Intermediate ISR: 67.7  No ISR: 67.8 | NA | ISR: 35.0  Intermediate ISR: 29.3  No ISR: 30.7 | Intracranial artery stenting implantation | Intracranial arteries (Internal carotid artery, Middle cerebral artery, vertebral artery, basilar artery) |

**S8a Table. Predictive Accuracy Data**

| **Author, Year** | **Blood Work Up Timing** | **Cut-off Value (Ratio)** | **Sensitivity (%)** | **Specificity (%)** | **AUC (95%CI)** |
| --- | --- | --- | --- | --- | --- |
| Balli et al | Post Procedural | 3.43 | 0.455 | 0.958 | 0.771 (0.703-0.830) |
| Bolca et al | Pre Procedural | 3.84 | 0.734 | 0.508 | 0.604 |
| Chang et al | Pre Procedural | 3.62 | 0.734 | 0.804 | 0.707 (0.603-0.792) |
| Lee et al | Post Procedural | 2.75 | 0.813 | 0.508 | 0.632 |
| Li et al | Pre Procedural | 3.16 | 0.754 | 0.721 | 0.820 (0.770-0.870) |
| Pan et al | Post-Procedural | 2.78 | 0.808 | 0.532 | 0.666 (0.541-0.791) |
| Song et al | Pre Procedural | NA | 0.710 | 0.624 | 0.707 (0.627-0.788) |
| Turak et al | Pre Procedural | 2.73 | 0.800 | 0.750 | 0.850 (0.810-0.900) |
| Wang et al | Pre-Procedural | 2.51 | 0.750 | 0.700 | 0.800 (0.710-0.890) |
| Yang et al | Post Procedural | 4.3 | 0.750 | 0.760 | 0.779 (0.710-0.840) |

**S8b Table. Prognostic Test Data**

| **Author, Year** | **Blood Work-up Timing** | **Adjusted Variables** | **Odds Ratio (95%CI)** | |
| --- | --- | --- | --- | --- |
|  |  |  | **Cut-off (Ratio)** | **Adjusted** |
| Balli et al, 2015 | Post Procedural | Age, DM, Sex, LVEF, Main branch inflation pressure (atm), Main branch stent diameter (mm), Main branch stent length (mm), Pre-intervention main branch stenosis degree %, Pre-intervention side branch stenosis degree %, Side branch inflation pressure (atm), Side branch stent diameter (mm), Side branch stent length (mm) | 3.43 | 6.98 (3.19-15.27) |
| Bolca et al, 2015 | Pre Procedural | Creatinine, CRP, LDL, Pre-PCI TIMI 0 flow, Sex, Stent diameter, Stent length, Stent method | /unit | 1.13 (1.08-1.19) |
| Chang et al, 2018 | Pre Procedural | CRP, DM, Hyperlipidemia, Mean lesion length, Number of runoff vessels | 3.62 | 1.70 (1.52-2.06) |
| Dai et al, 2019 | Pre Procedural | Residual Stenosis, Serum Glucose, Smoking | 2.13 | 2.74 (1.46-5.14) |
| Gabbasov et al, 2018 | Pre Procedural | Arterial diameter <2.75 mm, CD45+ platelet, DM, Number of stents | /unit | 1.61 (1.15-2.25) |
| Lee et al, 2020 | Post Procedural | Diabetes Mellitus, Hyperlipidemia, Hypertension, Smoking | 2.75 | 3.10  (1.3-7.7) |
| Li et al, 2019 | Pre Procedural | Diabetes mellitus, Dissection, eGFR, hsCRP, Low-density lipoprotein, PLR, RDW, Smoker, Stent Length, Total cholesterol | /unit | 1.7  (1.43-2.01) |
| Luo et al, 2022 | Pre Procedural | COPD, GS Scores, Monocyte, Remnant Cholesterol | /unit | 1.11 (1.01-1.21) |
| Pan et al, 2022 | Post Procedural | Age, Coronary artery disease, Critical limb ischemia, Diabetes Mellitus, De novo lesion, Hypertension, Lesion length, Male, Popliteal involvement, Severe calcification, Smoking, TASC C/D, Total occlusion | /unit | 1.40 (1.07-1.84) |
| Song et al, 2024 | Pre Procedural | Age, CHA2DS2-VASc score, CHA2DS2-VASc risk score, Congestive heart failure, LVEF (%), PLR, Prior stroke | /unit | 1.22 (1.02-1.46) |
| Turak et al, 2012 | Pre Procedural | CRP, Current smoker, DM, HDL, LDL, LVEF, Stent diameter, Stent length, TG, Time between 2 coronary angiographies | 2.73 | 1.85 (1.13-2.45) |
| Wang et al, 2020 | Pre Procedural | Diabetes, hs-CRP, Lesion length, LDL-C, Rivaroxaban | /unit | 1.47 (1.13-2.48) |
| Yang et al, 2020 | Post Procedural | DM, LDL, Post-interventional lymphocyte count, Post-interventional monocyte count, Post-interventional PDW, Pre-interventional PDW | 4.3 | 1.95 (1.51-2.50) |
| Yu et al, 2022 | Pre Procedural | Age, BMI, CAD, Concurrent intracranial tandem stenosis, Diabetes mellitus, Fibrinogen, Fasting plasma glucose, hs-CRP (>3mg/L), Hyperlipidemia, Lesion length, Male, Mori types, Residual stenosis | /unit | 1.48 (1.06-2.04) |

**S9 Table. Baseline Characteristics of Included Studies.**

| **Author** | **Study Period** | **Follow-up Period** | **Study Design** | **Study Location** | **Laboratorium Brand** | **Population** | **Sample Size (Man %)** | **Age** | **Comorbidities** | | | | | **Procedure**  **(Type of Stent)** | **Stent Location** |
| --- | --- | --- | --- | --- | --- | --- | --- | --- | --- | --- | --- | --- | --- | --- | --- |
|  |  |  |  |  |  |  |  |  | **HT (%)** | **DM (%)** | **DL (%)** | **AF (%)** | **Smoking (%)** |  |  |
| Balli et al et al., 2015 | January 2010 - December 2012 | ISR (+): 9.6 ± 3.4  ISR (-): 8.9 ± 3.9 | Cohort Prospective* | Turkey | NA | Stable CAD patients with true bifurcation lesion | 181 (61.6) | ISR: 55.3 ± 10.0, non ISR: 56.4 ± 12.4 | ISR: 52.3  No ISR: 61.3 | ISR: 38.6  No ISR: 22.6 | ISR: 70.5  No ISR: 73.1 | NA | NA | Percutaneous Coronary Intervention  (Bare-metal stent) | Bifurcation area of the coronary arteries |
| Bolca et al., 2015 | January 2008 - December 2012 | 14 (6-60) | Cohort Retrospective | Turkey | Coulter LH 780 Hematology Analyzer; Beckman Coulter Ireland Inc., Mervue, Galway, Ireland | ST-segment elevation myocardial infarction (STEMI) patients and had undergone primary PCI | 404 (66.34) | Tertile 1: 14.5 ± 1.2  Tertile 2: 14.6 ± 1.2  Tertile 3: 14.4 ± 1.1 | Tertile 1: 45.0  Tertile 2: 39.0  Tertile 3: 44.0 | Tertile 1: 22.0  Tertile 2: 25.0  Tertile 3: 22.0 | Tertile 1: 36.0  Tertile 2: 30.0  Tertile 3: 30.0 | NA | Tertile 1: 35.0  Tertile 2: 50.0  Tertile 3: 61.0 | Percutaneous Coronary Intervention  (Bare-metal stent) | Coronary arteries (Left circumflex artery, left anterior descending artery, right coronary artery) |
| Chang et al., 2018 | January 2012 - December 2014 | 12 months | Cohort Prospective | China | Automated Blood Cell Counter (Model XE2100; Sysmex Co, Kobe, Japan) | Femoropopliteal chronic total occlusions patients | 180 (78) | ISR: 64.1 ± 11.0, Non-ISR: 68.0 ± 11.8 | ISR: 60.5  No ISR: 60.5 | ISR: 78.9  No ISR: 41.1 | ISR: 84.2  No ISR: 54.0 | NA | ISR: 44.7  No ISR: 47.6 | Percutaneous Coronary Intervention  (Bare-metal stent) | Femoral and popliteal artery |
| Dai et al., 2019 | March 2004 - December 2016 | 14.6 ± 19.1 (range:  0.7-120.7) months | Cohort Prospective | China | Automated Blood Analyzer (Sysmex XE-2100, Kobe, Japan) | Carotid artery stenosis | 295 (85.8) | ISR: 66.6 ± 7.1, non ISR: 66.9 ± 7.6 | 82.8 | 37.0 | 44.0 | NA | 39.2 | Carotid angioplasty and stenting  (Self-expandable stent) | Carotid artery |
| Gabbasov et al., 2018 | January 2012 - December 2015 | 6-12 months | Cohort Prospective | Russia | NA | Patients With Diabetes  Having Stable Coronary Artery Disease | 126 | DM: 62.4 (9.5)  Non-DM: 62.2 (11.3) | DM: 98.2  Non-DM: 91.2 | NA | DM: 100.0  Non-DM: 98.6 | NA | DM: 50.9  Non-DM: 47.9 | Percutaneous Coronary Intervention  (Drug-eluting stent) | Coronary arteries (Left circumflex artery, left anterior descending artery, right coronary artery) |
| Lee et al., 2020 | NA | 24 months | Cohort Prospective | Austria | NA | Infrainguinal artery stenosis of PAD with Rutherford stage 2-3 patients | 95 (60) | 65 (58-74) | 92.6 | 35.8 | 93.7 | NA | 44.2 | Infrainguinal angioplasty with stent  Implantation  (N/A) | Superficial femoral artery |
| Li et al., 2019 | January 2010 - April 2013 | 14.4 ± 3.3 months | Cohort Retrospective | China | Sysmex XS 500i auto analyzer (Sysmex,  Kobe, Japan) | Coronary chronic total occlusion (CTO) | 416 | ISR: 57.8 ± 10.5, non ISR: 60.2 ± 11.7 | CTO: 58.8  Non-CTO: 45.0 | CTO: 37.5  Non-CTO: 34.4 | CTO: 60.0  Non-CTO: 50.6 | NA | CTO: 58.1  Non-CTO: 34.4 | Percutaneous coronary intervention  (Drug-eluting stent) | Coronary arteries (Left circumflex artery, left anterior descending artery, right coronary artery) |
| Luo et al., 2022 | January 2017 and December 2020 | 6-24 months | Cohort Retrospective | China | NA | Patients with coronary artery disease | 477 (79) | ISR: 63(55.7)  non-ISR: 61(54.7) | ISR: 48.9  No ISR: 49.2 | ISR: 19.1  No ISR: 15.1 | NA | NA | ISR: 62.8  No ISR: 55.9 | Percutaneous coronary intervention  (Drug-eluting stent) | Coronary arteries (Left circumflex artery, left anterior descending artery, right coronary artery) |
| Pan et al., 2023 | January 2017 and December 2019 | Every 3-6 months (1, 3, 6 months, and so on) | Cohort Retrospective | China | Model XE2100; Sysmex Co, Kobe, Japan | Femoropopliteal arterial disease | 120 (78) | 70.0 ± 8.3 | Non-restenosis: 70.3  Early restensosis: 55.0  Late restenosis: 65.4 | Non-restenosis: 59.5  Early restensosis: 80.0  Late restenosis: 57.6 | NA | NA | Non-restenosis: 45.9  Early restensosis: 20.0  Late restenosis: 50.0 | Drug-coated balloon (DCB) angioplasty | Femoral and popliteal artery |
| Song et al., 2024 | January 2017 and January 2022 | 24 months | Cohort Retrospective | China | NA | Severe kidney disease (eGFR < 30 ml/min 1.73m) receiving coronary stent placement | 164 | ISR: 72 (70.6)  Non-ISR: 35 (56.5) | ISR: 95.2  Non-ISR: 85.3 | ISR: 67.4  Non-ISR: 54.9 | ISR: 87.1  Non-ISR: 87.3 | ISR: 6.5  Non-ISR: 6.9 | ISR: 22.6  Non-ISR: 33.3 | Percutaneous coronary intervention  (N/A) | Coronary arteries (Left circumflex artery, left anterior descending artery, right coronary artery) |
| Turak et al., 2012 | February 2008 - June 2010 | N/A | Cohort Retrospective | Turkey | Automated blood cell counter (ADVIA 2120i Hematology System, Siemens Healthcare Diagnostics, Deerfield, Illinois) | Stable or Unstable Angina Pectoris Patients | 624 (72) | 60.5 ± 10.2 | Tertile 1: 53.0  Tertile 2: 59.0  Tertile 3: 62.0 | Tertile 1: 28.0  Tertile 2: 30.0  Tertile 3: 35.0 | NA | NA | Tertile 1: 37.0  Tertile 2: 43.0  Tertile 3: 55.0 | Percutaneous Coronary Intervention  (Bare-metal stent) | Coronary arteries (Left circumflex artery, left anterior descending artery, right coronary artery) |
| Wang et al., 2022 | May 2016 to December 2020 | 12 months | Cohort Retrospective | China | NA | Lower extremity arterial ischemia symptoms and Rutherford grade 3-5 | 117 (66) | 67.0 (59.0, 71.0) | 41.0 | 65.0 | NA | NA | 10.3 | Drug-coated balloon angioplasty | Femoral and popliteal artery |
| Wang et al | January 2016 and January 2019 | 6-15 months | Cohort Retrospective | China | NA | Coronary artery disease | 96 (71) | 65.3 ± 8.4 | NA | NA | NA | NA | NA | Percutaneous coronary intervention  (Drug-eluting stent) | Coronary arteries (Left circumflex artery, left anterior descending artery, right coronary artery) |
| Yang et al | March 2015 - July 2018 | 12 months | Cohort Prospective | China | Auto-Analyzer (Model XE2100; Sysmex Co, Kobe, Japan) | Lower extremity arteriosclerosis obliterans patients | 199 (77.9) | ISR: 68.72 ± 11.37, Non-ISR: 70.59 ± 9.52 | ISR: 77.5  No ISR: 82.4 | ISR: 42.5  No ISR: 32.6 | ISR: 80.0  No ISR: 67.8 | NA | ISR: 35.0  No ISR: 30.7 | Percutaneous transluminal stent implantation  (Self-expanding balloon) | Superficial femoral artery |
| Yu et al | June 2012 to September 2019 | 48 months | Cohort Retrospective | China | Automatic particle counters at hematology laboratories in Tiantan Hospital | Symptomatic intracranial atherosclerotic stenosis | 279 | 57.73 ± 8.73 | ISR: 77.5  Intermediate ISR: 76.7  No ISR: 82.4 | ISR: 42.5  Intermediate ISR: 37.1  No ISR: 32.7 | ISR: 80.0  Intermediate ISR: 67.7  No ISR: 67.8 | NA | ISR: 35.0  Intermediate ISR: 29.3  No ISR: 30.7 | Intracranial artery stenting implantation | Intracranial arteries (Internal carotid artery, Middle cerebral artery, vertebral artery, basilar artery) |

**S10 Table. Outcomes of NLR Predictive Value for ISR**

| **Author, Year** | **Blood Work Up Timing** | **Cut-off Value (Ratio)** | **Sensitivity (%)** | **Specificity (%)** | **AUC (95%CI)** |
| --- | --- | --- | --- | --- | --- |
| Balli et al | Post Procedural | 3.43 | 0.455 | 0.958 | 0.771 (0.703-0.830) |
| Bolca et al | Pre Procedural | 3.84 | 0.734 | 0.508 | 0.604 |
| Chang et al | Pre Procedural | 3.62 | 0.734 | 0.804 | 0.707 (0.603-0.792) |
| Lee et al | Post Procedural | 2.75 | 0.813 | 0.508 | 0.632 |
| Li et al | Pre Procedural | 3.16 | 0.754 | 0.721 | 0.820 (0.770-0.870) |
| Pan et al | Post-Procedural | 2.78 | 0.808 | 0.532 | 0.666 (0.541-0.791) |
| Song et al | Pre Procedural | NA | 0.710 | 0.624 | 0.707 (0.627-0.788) |
| Turak et al | Pre Procedural | 2.73 | 0.800 | 0.750 | 0.850 (0.810-0.900) |
| Wang et al | Pre-Procedural | 2.51 | 0.750 | 0.700 | 0.800 (0.710-0.890) |
| Yang et al | Post Procedural | 4.3 | 0.750 | 0.760 | 0.779 (0.710-0.840) |

**S11 Table. NLR and Prognosis of ISR**

| **Author, Year** | **Blood Work-up Timing** | **Adjusted Variables** | **Odds Ratio (95%CI)** | |
| --- | --- | --- | --- | --- |
|  |  |  | **Cut-off (Ratio)** | **Adjusted** |
| Balli et al, 2015 | Post Procedural | Age, DM, Sex, LVEF, Main branch inflation pressure (atm), Main branch stent diameter (mm), Main branch stent length (mm), Pre-intervention main branch stenosis degree %, Pre-intervention side branch stenosis degree %, Side branch inflation pressure (atm), Side branch stent diameter (mm), Side branch stent length (mm) | 3.43 | 6.98 (3.19-15.27) |
| Bolca et al, 2015 | Pre Procedural | Creatinine, CRP, LDL, Pre-PCI TIMI 0 flow, Sex, Stent diameter, Stent length, Stent method | /unit | 1.13 (1.08-1.19) |
| Chang et al, 2018 | Pre Procedural | CRP, DM, Hyperlipidemia, Mean lesion length, Number of runoff vessels | 3.62 | 1.70 (1.52-2.06) |
| Dai et al, 2019 | Pre Procedural | Residual Stenosis, Serum Glucose, Smoking | 2.13 | 2.74 (1.46-5.14) |
| Gabbasov et al, 2018 | Pre Procedural | Arterial diameter <2.75 mm, CD45+ platelet, DM, Number of stents | /unit | 1.61 (1.15-2.25) |
| Lee et al, 2020 | Post Procedural | Diabetes Mellitus, Hyperlipidemia, Hypertension, Smoking | 2.75 | 3.10  (1.3-7.7) |
| Li et al, 2019 | Pre Procedural | Diabetes mellitus, Dissection, eGFR, hsCRP, Low-density lipoprotein, PLR, RDW, Smoker, Stent Length, Total cholesterol | /unit | 1.7  (1.43-2.01) |
| Luo et al, 2022 | Pre Procedural | COPD, GS Scores, Monocyte, Remnant Cholesterol | /unit | 1.11 (1.01-1.21) |
| Pan et al, 2022 | Post Procedural | Age, Coronary artery disease, Critical limb ischemia, Diabetes Mellitus, De novo lesion, Hypertension, Lesion length, Male, Popliteal involvement, Severe calcification, Smoking, TASC C/D, Total occlusion | /unit | 1.40 (1.07-1.84) |
| Song et al, 2024 | Pre Procedural | Age, CHA2DS2-VASc score, CHA2DS2-VASc risk score, Congestive heart failure, LVEF (%), PLR, Prior stroke | /unit | 1.22 (1.02-1.46) |
| Turak et al, 2012 | Pre Procedural | CRP, Current smoker, DM, HDL, LDL, LVEF, Stent diameter, Stent length, TG, Time between 2 coronary angiographies | 2.73 | 1.85 (1.13-2.45) |
| Wang et al, 2020 | Pre Procedural | Diabetes, hs-CRP, Lesion length, LDL-C, Rivaroxaban | /unit | 1.47 (1.13-2.48) |
| Yang et al, 2020 | Post Procedural | DM, LDL, Post-interventional lymphocyte count, Post-interventional monocyte count, Post-interventional PDW, Pre-interventional PDW | 4.3 | 1.95 (1.51-2.50) |
| Yu et al, 2022 | Pre Procedural | Age, BMI, CAD, Concurrent intracranial tandem stenosis, Diabetes mellitus, Fibrinogen, Fasting plasma glucose, hs-CRP (>3mg/L), Hyperlipidemia, Lesion length, Male, Mori types, Residual stenosis | /unit | 1.48 (1.06-2.04) |

**S1 Figure. PRISMA Checklist**


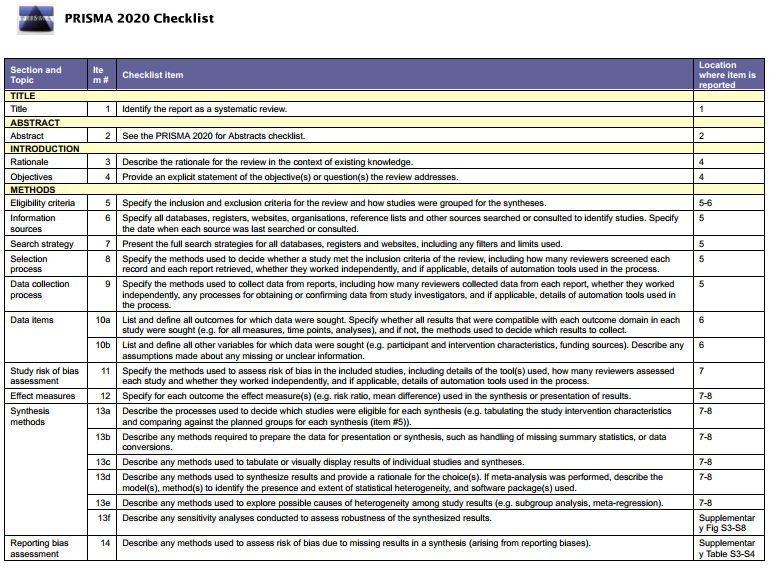


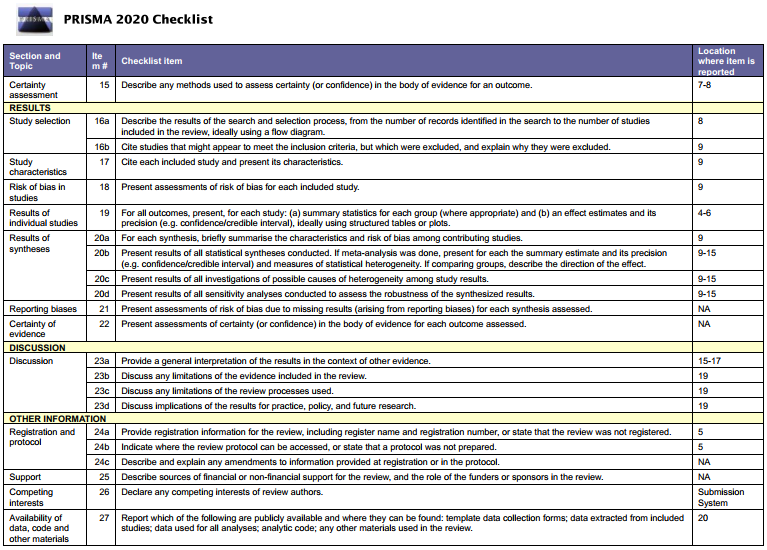


**S2 Figure. Leave-One-Out Sensitivity Analysis**


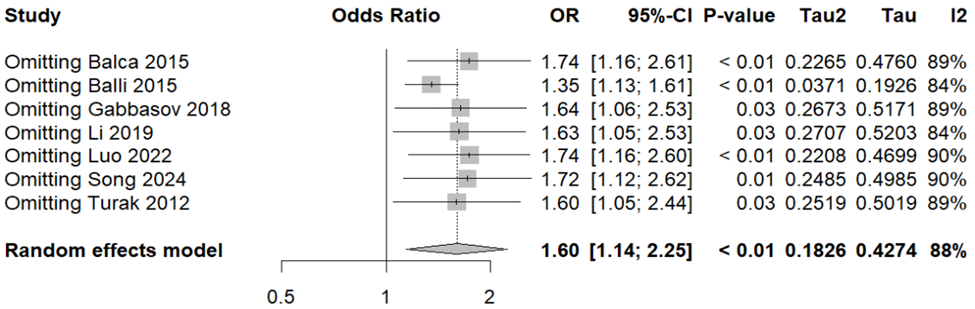


**S3 Figures. Subgroup Analysis Forest Plots** **According to Study Design**


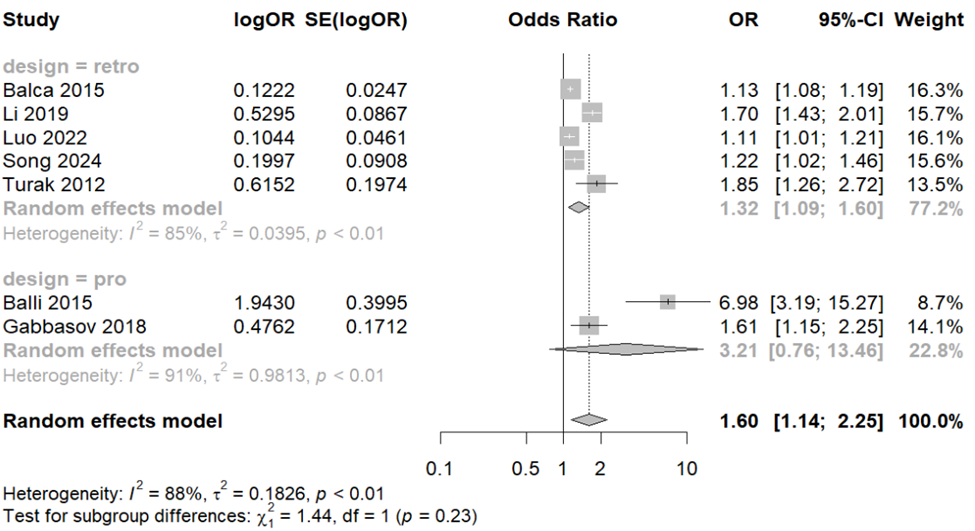


**S4 Figures. Subgroup Analysis Forest Plots According to Region**


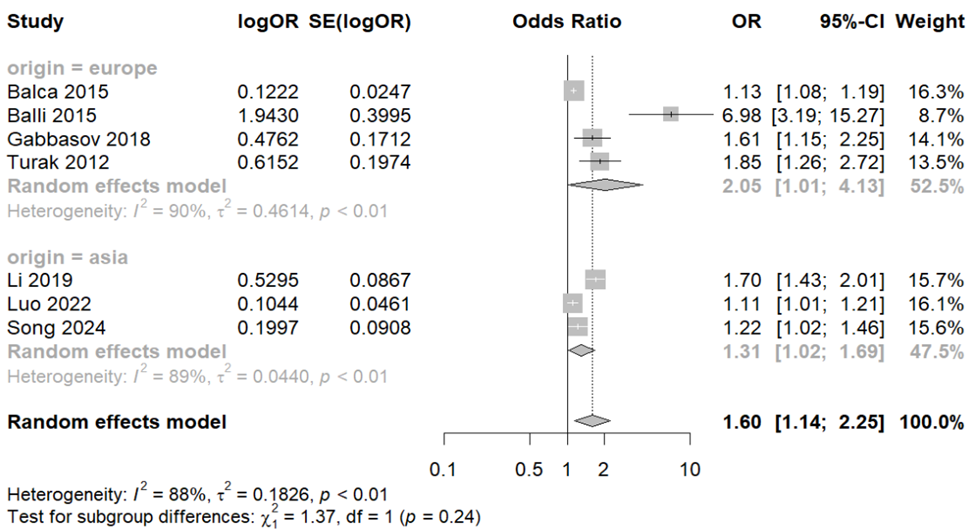


**S5 Figures. Subgroup Analysis Forest Plots According to Dose Category**


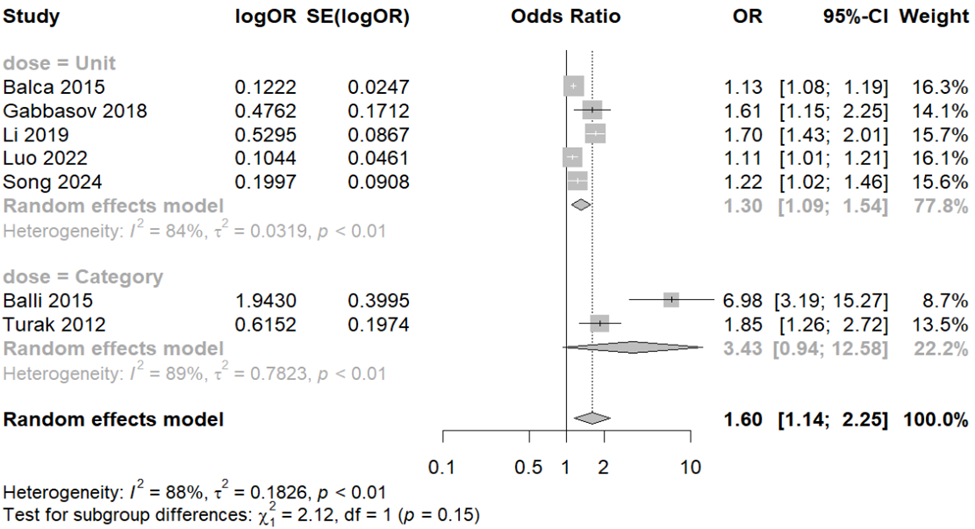


**S6 Figures. Subgroup Analysis Forest Plots According to Sample Size**


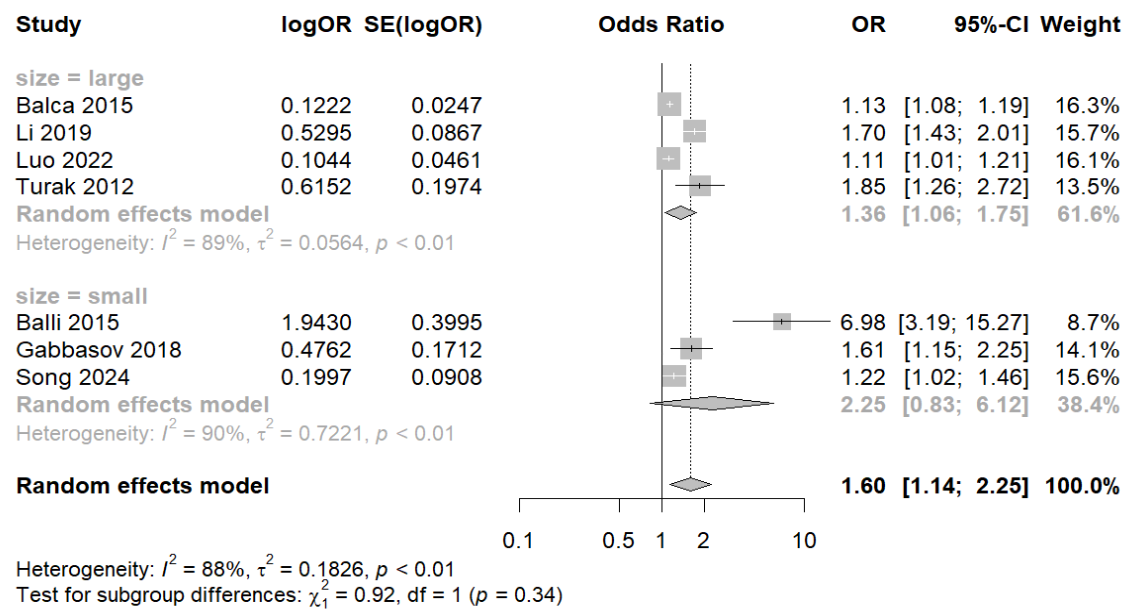


**S7 Figures. Subgroup Analysis Forest Plots According to Follow-up Period**


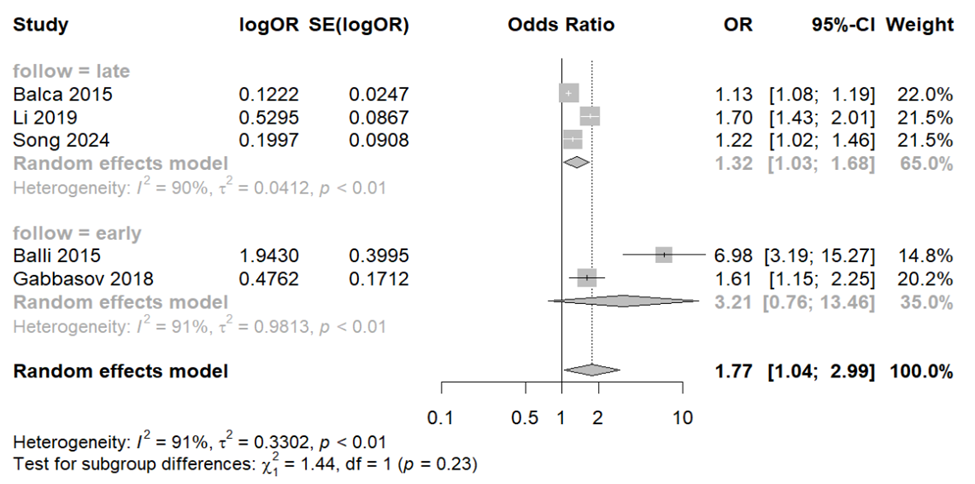


**S8 Figures. Linear Dose-Response Relationships between NLR and Coronary ISR**


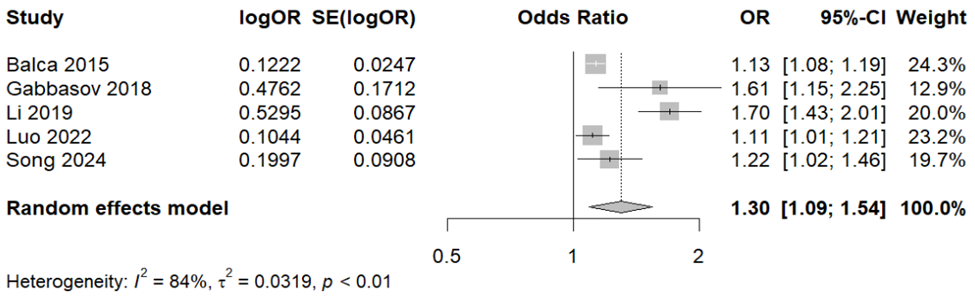


**S9 Figures. Linear Dose-Response Relationships between NLR and Non-coronary ISR**


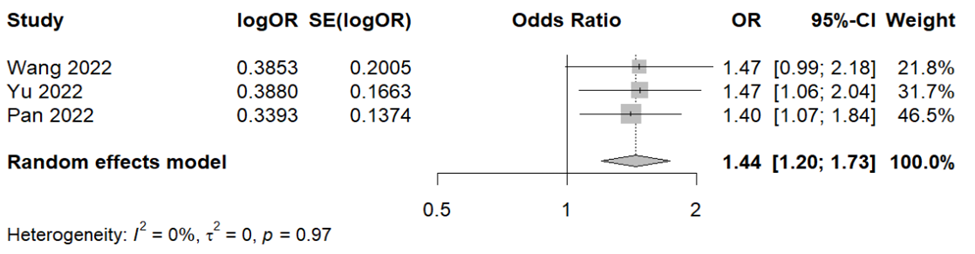

Supplement: S1 File — (DOCX) [file pone.0322461.s001.docx]
